# Developing Image Processing Meta-Algorithms with Data Mining of Multiple Metrics

**DOI:** 10.1155/2014/383465

**Published:** 2014-02-05

**Authors:** Kelvin Leung, Alexandre Cunha, A. W. Toga, D. Stott Parker

**Affiliations:** ^1^Intel Corporation, 3600 Julliette Ln., Mail Stop SC12-301, Santa Clara, CA 95054, USA; ^2^UCLA Computer Science Department, Los Angeles, CA 90095-1596, USA; ^3^Caltech Center for Advanced Computing Research (CACR), Pasadena, CA 91125, USA; ^4^USC Laboratory of Neuroimaging (LONI), Los Angeles, CA 90007, USA

## Abstract

People often use multiple metrics in image processing, but here we take a novel approach of mining the values of batteries of metrics on image processing results. We present a case for extending image processing methods to incorporate automated mining of multiple image metric values. Here by a metric we mean any image similarity or distance measure, and in this paper we consider intensity-based and statistical image measures and focus on registration as an image processing problem. We show how it is possible to develop meta-algorithms that evaluate different image processing results with a number of different metrics and mine the results in an automated fashion so as to select the best results. We show that the mining of multiple metrics offers a variety of potential benefits for many image processing problems, including improved robustness and validation.

## 1. Introduction

Every year many articles are published in the area of biomedical image registration that introduce new metrics for biomedical images, covering both distance/difference measures and similarity measures. There are many reasons for this interest in metrics. However, the abundance of methods creates a basic dilemma for practitioners seeking high-performance imaging systems: which metric should be used?

This paper reports on an effort spanning five years at UCLA, studying this question, and developing schemes that use multiple methods and multiple evaluation metrics to obtain better image processing results. In much the same way that ensemble methods yield better results in data mining, this effort explored software combinations of metrics that yielded improved methods for registration in neuroimaging.

In this paper we consider two families of image similarity metrics: intensity-based metrics (metrics of the intensity or luminosity values of voxels) and statistical metrics (metrics of their distributions). There are at least three reasons why use of multiple metrics can be important in image processing as follows.

- Metrics are performance measures, so awareness of them is a prerequisite for good performance. Although it is common to commit *ab initio* to a single registration algorithm and metric, algorithms and metrics differ significantly, and choices among them can have important consequences.
- There are inherent limits to image processing performance. From this perspective, image processing methods are little more than optimizers that rest on assumptions about prior distributions of images and validation as experimental verification of these distributions. However, if metric values can be treated as samples of prior distributions on performance measures, we can mitigate some of these limits.
- The key point of this paper is that the results of different image processing algorithms and parameter settings can be evaluated under multiple metrics, and the metric values can then be analyzed with data mining to identify the best results. The tracking of metric value results permits investigation of which image processing methods give better results for images from a given source. It also permits flexible on-demand analysis of arbitrary performance measures.

Every metric has strengths and weaknesses when applied to categories of image modalities. In fact, some metrics are designed for or biased towards specific categories and therefore cannot encompass some images in real-world applications; no algorithm can be better than the metric used to evaluate it. Equivalently, proper evaluation of the performance of an algorithm can require consideration of multiple metrics.

Having multiple metric values is also important for development of image *meta-processing,* processing that analyzes the results of diverse algorithms, parameter settings, and metrics. In this paper we consider the use of a meta-algorithm in image registration, but the approach can be applied with many image processing algorithms. Data mining methods permit identification of relationships across algorithms and metrics.

## 2. Image Metrics

If *ℛ* and *𝒮* are two images we wish to compare, we compute a measure *D*(*ℛ*, *𝒮*), where *D* is a measure of similarity that we refer to as a *metric*. Although there are many metrics [1–3], the similarity between images either is commonly defined as a function of the intensities (luminosities) or intensity distributions of corresponding voxels across images or is based on the morphology of the features present in both images.

Every year many articles are published in the area of medical image registration that introduce new metrics. In the ideal case this multitude of options could be condensed into a set of metrics that are effective, comprehensive, and compact. We have implemented an initial approximation of this ideal. The metrics we consider here can be broadly divided into intensity-based metrics, which rely solely on the intensities of voxels, and statistical metrics, which are based on distributions of these intensities. These are simple and there are many others, but our implementation is open and representative and can in principle accommodate any metric.

Table 2 lists a few basic metrics. A survey covering the derivation and use of entropy-related metrics is in [4], and the Correlation metric and Woods metrics are summarized in [5]. Throughout this list, *N* is the size of the images (total number of voxels), and *x* ranges over the set of image voxels.

Metrics often depend on the application itself and on the modalities of the input images. Both intensity- and morphology-based metrics have been largely employed in the implementation of registration algorithms to attend different needs including comparing images with different modalities.

**Figure 1:**
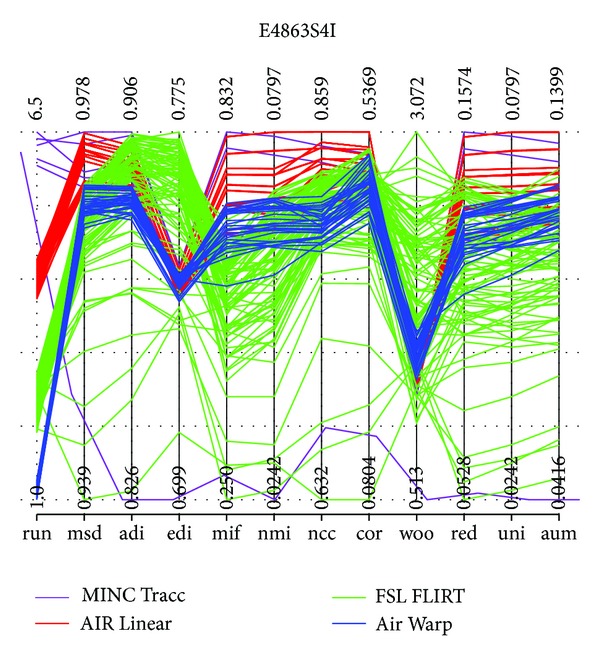
This parallel coordinates plot is a visual representation of our eleven metric values for 186 different variants of the image E4863S4I, produced by four image registration tools, AIR Warp (blue), AIR Linear (red), FSL FLIRT (green), and MINC Tracc (purple). Higher metric values are “better” (higher similarity or lower distance). Each trajectory across the plot gives the row of 11 metric values obtained by an image; altogether there are 186 such trajectories, so an entire table of 186 × 11 metric values is plotted here. Higher scores are better, but the results of the metric/score computations in each column have been independently scaled, so the columns represent different real intervals; the spread of the vertical axis values is not as dramatic as it may appear. Notice that some disorder occurs for the *edi* (Entropy of Difference of Intensities) and woo (Woods) metrics, but the ordering of results is otherwise fairly consistent across metrics.

The metrics in Table 2 illustrate how each metric has strengths and weaknesses when applied to categories of image modalities. Some metrics are designed for or biased towards specific categories and therefore cannot encompass all possible image types and qualities present in a given application. To permit comparison across metrics, we have forced all values to be scores in [0,1], with 1 being optimal.  Figure 1 shows all metric values for 186 variants of image E4863S4I produced by four registration tools.

## 3. Issues Raised by Use of Multiple Metrics

### 3.1. Metrics Measure Different Things and Can Be Inappropriate

There are many notions of similarity. This set of intensity-based and statistical metrics in Table 2 is *not* appropriate for some problems. For example, registration of images exhibiting neurodegeneration or brain trauma may yield counterintuitive results with these metrics and “better” metric values may not reflect more satisfactory alignment, since voxel-level measures may not capture global or semantic similarity. Metrics used should be suited to the problem.

Image metrics can involve image features (and therefore both feature detection and feature matching) as well as models (and therefore model estimation, image resampling, image transformation, and numerical optimization) [6]. Generally speaking, any aspect of image registration can be part of an image metric definition. These feature-based and model-based metrics can be compute-intensive, but the metrics in Table 2 do not impose heavy computational overhead.

### 3.2. Metrics Can Yield Inconsistent Results

**Figure 2:**
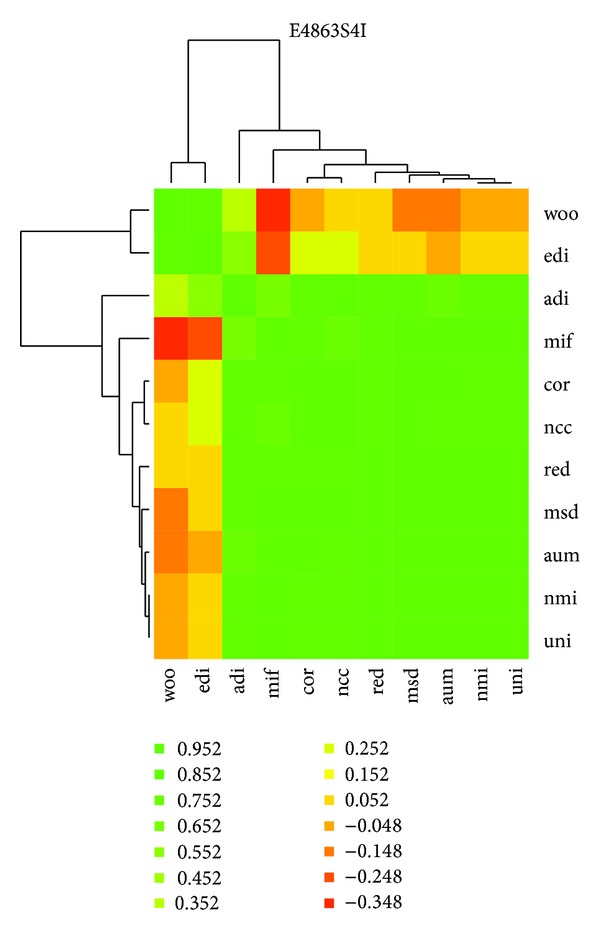
Heat map representation of the correlation matrix for the table of metric values for the 186 variants of input image E4863S4I, with metrics clustered into a hierarchy by rough similarity. The nine last metrics are consistent in the sense that they are highly correlated, with all pairwise correlation values above 0.644. There is nontrivial disagreement between these nine and the *edi* (Entropy of Difference of Intensities) and woo (Woods) metrics—also shown in Figure 1.

Consistency among these metrics can be visualized with a parallel coordinates plot of the data (Figure 1) or a visual representation of the correlation matrix (Figure 2) and thus the metric value table can be approximated by few dimensions. In this case, the *edi* metric is least consistent with the others, and this is reflected by the second principal component. More experience with this consistency may make it possible to analyze performance across families of metrics or develop theories concerning convex combinations of selected metrics. However, for dimensionality reduction to work the set of metrics have to be basically consistent, in the sense that their results have to be positively correlated. For example, the Woods metric [5] is given by

(1) $$\begin{array}{c} \text{woo}\left(ℛ,𝒮\right)=1-\frac{1}{N}\sum_{i}\frac{N\left(i\right)\sigma \left(i\right)}{\mu \left(i\right)},‍ \end{array}$$

where the index *i* ranges over intensity values, *N*(*i*) is the number of voxels in *ℛ* having value *i*, and *μ* and *σ* are the mean and standard deviation of intensities in *𝒮*, in the same voxel positions. In our experience this metric has often been anticorrelated with the other metrics. Although often well-suited to medical image registration problems, its inconsistency implies that the Woods metric can often yield very different results than other metrics.

Since metrics can be computed automatically, evaluating a set of them gives us not only an inexpensive way of assessing multiple aspects of similarity but also a strategy for eliminating poor results and a basis for machine learning. Automation will never eliminate the need for expert opinion, but it can help eliminate distractions and improve productivity.

### 3.3. Metric Values Can Be Stored in a Database for Analysis

In the course of this development we have refined its implementation, in the choice of metrics, in recording of results (with a database), and in various performance enhancements for increasing parallelism and reducing file movement. Specifically, we used a database system to record all metric values obtained by each run and also metadata about program execution. This permits use of other data analysis tools for evaluating the resulting tables of metric values and execution information.

In our implementation, a backend PostgreSQL database is used to record all metric values for later analysis. Using a database to store this information provides three important benefits. First, it endows our meta-algorithm with the ACID properties (atomicity, consistency, isolation, and durability) provided by database systems. This is significant in a world in which tools crash or are unreliable as is unfortunately the case in neuroimaging. It might be possible to provide some of these properties in an ad hoc way, but there is little apparent gain in reimplementing these hard-won database features. Second, it allows our meta-algorithm to operate effectively in parallel computing environments. Using a database to log results independently is an elegant way to meet this need. Third, it allows ad hoc extraction and analysis of data from these executions. Although a given set of executions may not be that large (186 runs in our example), having a database makes this information much easier to work with.

Managing information about metric values in a database presents interesting possibilities for data mining. For example, one not only can determine which algorithms and parameter settings give better results for images from a given source, but also analyze execution times and even differences in performance by different versions of a given algorithm.

## 4. Developing Meta-Algorithms for Image Processing with Data Mining of Multiple Metrics

We show in this section how image processing methods can be extended by augmenting them with multiple metric computation coupled with data analysis methods from machine learning and data mining. As mentioned earlier, tracking metric value information (such as in a database) permits investigation of which algorithms and parameter settings give better results for images from a given source and permits analysis of execution times and even differences in performance by different versions of a given algorithm.

### 4.1. Evaluating Image Processing Methods with Multiple Metrics

Augmentation with metric evaluation is a natural evolutionary direction for image processing methods. Given a set of images *𝒮* = {*𝒮*_1_,…, *𝒮*_*n*_} (produced possibly with different methods or parameter settings and possibly with different input images), we can evaluate the similarity of an image *ℛ* with each *𝒮*_*i*_ ∈ *𝒮* under a battery of metrics *D*_*j*_, *j* = 1,…, *p*. The result of evaluation is then a *n* × *p* table *M* = (*m*_*ij*_), whose *ij*th entry is *m*_*ij*_ = *D*_*j*_(*ℛ*, *𝒮*_*i*_). With the *p* = 11 metrics in Table 2, *M* is a *n* × 11 table of metric values.

Image processing methods can then be augmented with a final data analysis phase. This analysis can yield deeper understanding of method under the various metrics. As long as performance can be formalized in terms of metrics, we believe that this extension with learning and data mining methods can be important in improving any scientific computational method, because it can rise above assumptions about input data that are tacit in development.

### 4.2. Example Application: Image Registration

Essentially, image registration is the problem of aligning two images. Since this alignment generally requires measurement of image similarity and optimization of a transformation so as to maximize it, registration is a canonical image processing problem requiring the consideration of multiple metrics.

Let *ℛ* and *𝒯* be, respectively, the reference and template images we want to register. In image registration we typically look for a transformation *f* that minimizes *D*(*ℛ*, *f*(*𝒯*)), where *D* is a measure of distance between a pair of images. Thus, we want the transformed image *f*(*𝒯*) to be as close as possible to the target image *ℛ*. In general, if *D*(*ℛ*, *𝒮*)≃0, then we say *ℛ* and *𝒮* are similar. We want the mapping *f* to be homeomorphic so that points close together in one image are mapped to points close together in the other image. Also in principle *f* should have a continuous inverse satisfying *D*(*f* ^−1^(*ℛ*), *𝒯*) = *D*(*ℛ*, *f*(*𝒯*)), although in practice this requirement is weakened [6].

When assessing registration, it is natural to investigate how the edges from the template image are mapped to the corresponding edges in the reference image. In good registrations the mapped and reference edges are perfectly superimposed or very close in shape and space. The same applies to surfaces in three dimensions. This is the morphological view of registration.

Registration also can be approached from an information-theoretic point of view where image intensities are viewed as probability distributions. The analysis of similarity between distributions and intensities governs assessment of how well a registration algorithm performs. This perspective is natural for medical imaging; using a collection of metrics is useful for assessing the quality of registration methods, taking distributions and luminosities into account.

### 4.3. IRMA: An Image Registration Meta-Algorithm

IRMA is a meta-algorithm for image registration that was developed with the metrics above in mind [7]. As an individual module in distributions of the LONI Pipeline environment [8], it produced the results shown in Figure 1.

Figure 1 shows aggregate results of registering a brain image using several algorithms. The four algorithms here include two—Linear and Warp (nonlinear)—from the AIR registration package [5, 9], FLIRT from Oxford's FSL package [10], and the Tracc program from McGill MNI's MINC package [11]. Many different method/parameter combinations are used, as shown in Table 1. These sets of parameters have been chosen based on experience with these algorithms. They produce 5 × 3 × 2 = 30 runs of AIR Linear, 3 × 30 = 90 runs of AIR Warp, 5 × 3 × 4 = 60 runs of FSL FLIRT, and 6 runs of MINC Tracc. Altogether these 186 registration runs required about 1.5 hours to complete on a lightly loaded grid.

The values of all metrics were computed for the result of each run, and the tabulated results are shown in Figure 1. Thus the plot highlights some interesting aspects of the relative performance of these methods. However, the values for each metric have been rescaled independently, so that the spread in metric values covers the entire vertical scale. Thus the plot highlights the relative ordering among metric values. Of course, little about the relative merit of the four algorithms can be determined from one registration problem.

Figure 1 shows metric values for the 186 registration results produced by IRMA for the image E4863S4I. They show dramatically that the four image registration algorithms considered are not robust, in the sense that small changes in their parameters can produce very different results. Experienced users are aware of this sensitivity to parameter values, and that good registration results can require effort to produce. Some of this sensitivity is due to the difficulty of formalizing registration as an optimization problem, given the facts that each of the many metrics is a possible objective function, and all algorithms make assumptions about the input data that might fail to hold.

Figure 4 presents actual images produced by IRMA for the image E4863S4I. These examples show that IRMA both can detect poor registration results and can be used to improve the robustness of registration for significant classes of input problems. Notice that in the cases shown the data is well-approximated by a one-dimensional projection; the data spreads out horizontally more than vertically. In the third and fourth row of the plots, the best results are outliers (relatively isolated points at the right) produced by AIR Warp; that is, for these images, the best results are significantly different from most results produced by other algorithms. Thus we see again that the algorithms are not robust: minor changes in parameters can produce not only much better results but also very different results.

**Figure 3:**
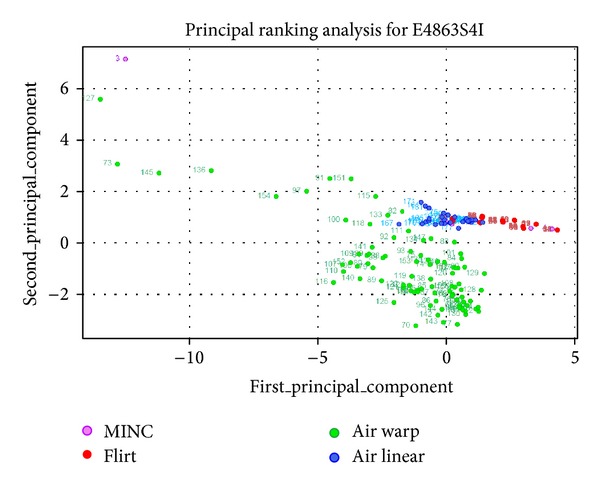
The table of metric values shown in Figure 1, after replacing metric values by their rankings, can be analyzed with principal component analysis (PCA). Replacement by rankings yields what is known as *robust* PCA, a nonparametric approach to dimensionality reduction with reduced sensitivity to outliers. The 11-dimensional metric value dataset is reduced here to a 2-dimensional plot along the first two principal components, showing that the FLIRT results (red points) generally dominate the others along the first principal component (*x*-axis). The AIR Warp results (green points) can dominate if we change the metric emphasis to the second principal component (*y*-axis).

**Figure 4:**
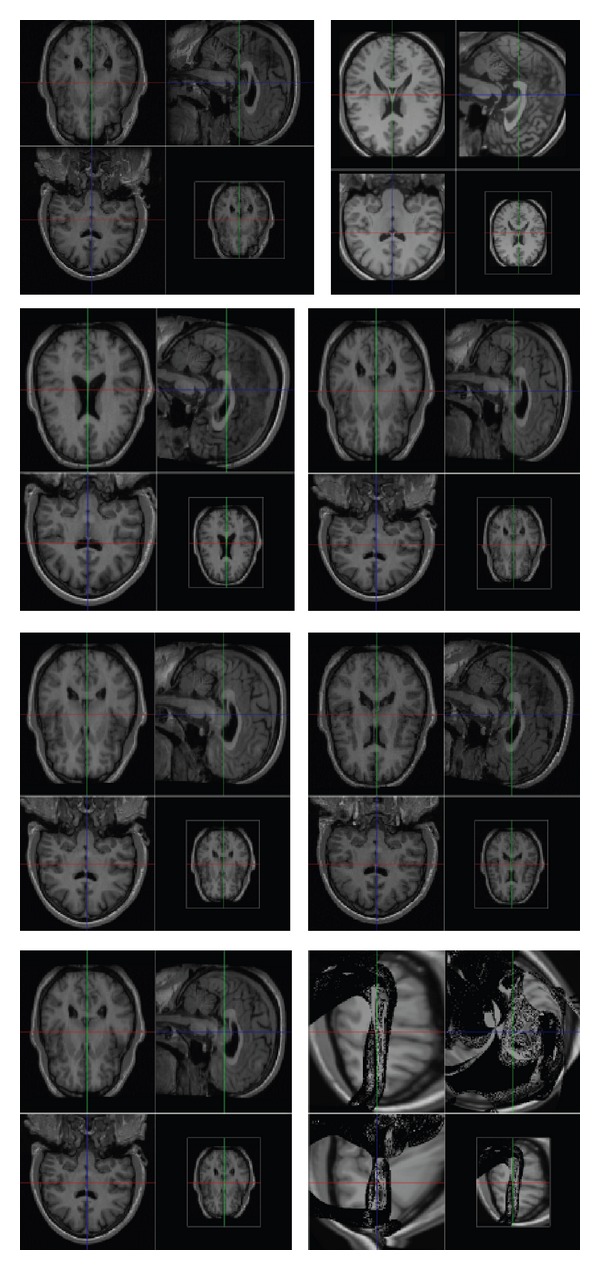
Some of the 186 registration results produced by IRMA for the input image E4863S4I, illustrating the wide variation in result quality that can be produced by changing algorithms and parameter settings. The first two images show the image projected into ICBM space (the target image) and the template (from the ICBM Atlas). Subsequent images (top-to-bottom, left-to-right) show the results produced by IRMA with ranks 1 (top ranked), 62, 88, 93, 124, and 186 (bottom ranked). Notice the significant diversity of result quality produced by different registration algorithms.

IRMA demonstrates how dimensionality reduction methods can be used to mine tables of metric values. Specifically, IRMA uses robust PCA [12, 13]—analyzing the principal components of Spearman rank correlation to extract latent ranking structure. As explained in Figure 1, differences in metric values are not necessarily as significant as the relative ordering among these values. Replacing the values in a dataset column by their relative ranking in that column removes scaling concerns and permits comparison of values across columns. Figures 1 and 3 show the result of making this nonparametric replacement. The clusters of results exhibit more structure, and the observations spread out more. In our experience this replacement can give useful perspective on the metric data. It also has the benefit that the covariance structure is identical to the correlation result, because the variance of each column is identical and known a priori.

Many dimensionality reduction methods are available—including alternative PCA methods and multidimensional scaling [13]. All these methods have potential applications with multiple metrics. Furthermore new meta-algorithm approaches could be developed using transformations of metric data.

If the performance of a given image processing method can be formalized in terms of the similarity metrics considered here, however, the multiple metric approach provides a more formal and more robust framework for validation. We can then extend the method to include a validation stage, which records computed metric values (e.g., in a database) and analyzes them (e.g., with PCA). Having multiple metrics as objectives formalizes them and avoids instabilities due to quirks of individual metrics.

By integrating data mining into our meta-algorithm we can increase sophistication of image processing algorithms. For example, IRMA's evaluation process can be extended to learn about the strengths and weaknesses of image processing methods and about the kinds of images encountered. IRMA also gains robustness from not relying on any single method or metric.

## 5. Conclusions

We have argued that many image processing methods can be beneficially extended to a meta-algorithm with standardized computation and data mining of multiple metric values. Although a metric can be any figure of merit, that is, useful in evaluating the performance of the method, we have considered the situation where each metric is an image similarity measure. In this approach, basic image processing algorithms are used to produce a collection of results (e.g., for a variety of alternative parameter settings); these results are evaluated with multiple metrics, and a data mining postprocessing phase is used to extract good results. The approach described here could lead to more formal and robust image processing methods that exploit machine learning, leading to better understanding of performance in many dimensions.

As a demonstration, in this paper we have described the IRMA image registration meta-algorithm. IRMA is a neuroimaging module in the LONI Pipeline workflow environment [14]. Image registration, the basic problem of aligning two images, rests fundamentally on the idea of a metric and immediately raises the issues discussed here about the choice of metrics. The ability to mine these data is consistent with learning methods and has compelling possibilities in fields like neuroimaging that involve many algorithms and diverse objectives. IRMA was developed with these possibilities in mind.

## Figures and Tables

**Table 1:** Parameter spaces of the four algorithms used in a representative configuration of IRMA, along with the resulting number of runs of each algorithm.

Algorithm	Options/parameters used	Runs
AIR warp	Airwarp model ∈{1,2, 3} and parameters for AIR linear	90
AIR linear	Blur *∈*{11,15,17,19,25}, model ∈{6,7, 9}, cost *∈*{1,3}	30
FSL FLIRT	Interpolation *∈* {trilinear, nearestnbr, sinc}, dof *∈*{6,7, 8,12}, cost *∈* {mutualinfo, corratio, normcorr, normmi, leastsq}	60
MINC Tracc	dof *∈*{3,6, 7,9, 10,12}	6

**Table 2:** Some intensity-based and statistical image metrics. In the Difference metrics, the index *x* ranges over voxel positions. In the Correlation and Woods metrics, the index *i* ranges over intensity values, *N*(*i*) is the number of voxels in *ℛ* having intensity *i*, and *μ*(*i*) and *σ* ^2^(*i*) are the mean and variance of intensities of *𝒮* in the same voxel positions. Normalized Cross-Correlation is voxel-wise correlation, with means and standard deviations computed over the entire image.

(1) *Mean Square Difference of Intensities *	
$\text{msd}(ℛ,𝒮)=\frac{1}{N}\underset{x}{\sum }{\left(ℛ\left(x\right)-𝒮\left(x\right)\right)}^{2}$	
(2) *Absolute Difference of Intensities *	
$\text{adi}(ℛ,𝒮)=\frac{1}{N}\underset{x}{\sum }\left|ℛ(x)-𝒮(x)\right|$	
(3) *Shannon Entropy of Difference of Intensities *	
$\text{edi}(ℛ,𝒮)=\frac{1}{N}\underset{x}{\sum }p\left(ℛ\left(x\right)-𝒮\left(x\right)\right)\log p\left(ℛ\left(x\right)-𝒮\left(x\right)\right)$	
(4) *Mutual Information *	
mif(*ℛ*, *𝒮*) = *ℐ*(*ℛ*, *𝒮*) = *ℋ*(*ℛ*) + *ℋ*(*𝒮*) − *ℋ*(*ℛ*, *𝒮*)	
(5) *Normalized Mutual Information *	
$\text{nmi}(ℛ,𝒮)=\frac{ℐ(ℛ,𝒮)}{ℋ(ℛ,𝒮)}+1=\frac{ℋ(ℛ)+ℋ(𝒮)}{ℋ(ℛ,𝒮)}$	
(6) *Normalized Cross-Correlation *	
$\text{ncc}(ℛ,𝒮)=\frac{\mathrm{cov}\left(ℛ,𝒮\right)}{\sigma_{ℛ}\sigma_{𝒮}}$	
(7) *Correlation *	
$\text{cor}\left(ℛ,𝒮\right)=1-\frac{1}{N}\underset{i}{\sum }\frac{N\left(i\right)\sigma^{2}\left(i\right)}{\sigma^{2}}$	
(8) *Woods *	
$\text{woo}\left(ℛ,𝒮\right)=1-\frac{1}{N}\underset{i}{\sum }\frac{N\left(i\right)\sigma \left(i\right)}{\mu \left(i\right)}$	
(9) *Redundancy *	
$\text{red}(ℛ,𝒮)=\frac{ℐ(ℛ,𝒮)}{\left(ℋ\left(ℛ\right)+ℋ\left(𝒮\right)\right)}=1-\frac{ℋ(ℛ,𝒮)}{\left(ℋ\left(ℛ\right)+ℋ\left(𝒮\right)\right)}$	
(10) *A Universal Metric *	
$\text{uni}(ℛ,𝒮)=1-\frac{ℐ\left(ℛ,𝒮\right)}{ℋ\left(ℛ,𝒮\right)}=2-\frac{\left(ℋ\left(ℛ\right)+ℋ\left(𝒮\right)\right)}{ℋ(ℛ,𝒮)}$	
(11) *Another Universal Metric *	
$\text{aum}(ℛ,𝒮)=1-\frac{ℐ\left(ℛ,𝒮\right)}{\max\left(ℋ\left(ℛ\right),ℋ\left(𝒮\right)\right)}$	
